# What's new in emergencies, trauma, and shock? JETS policy for publishing animal studies

**DOI:** 10.4103/0974-2700.50738

**Published:** 2009

**Authors:** Veronica Tucci, Sagar Galwankar, Tracy Sanson, Kelly O'Keefe

**Affiliations:** University of South Florida College of Medicine, Tampa, Florida, USA

Physician-scientists have utilized animal research to advance the state of medical art since virtually the beginning of recorded history.

Galen, an ancient Roman physician and philosopher, dissected pigs, apes, and other animals to further his understanding of human anatomy and physiology as the dissection of human corpses was against Roman law. More recent examples of physicians using animals to understand the workings of the human body include Drs. Frederick Banting and John Macleod who used dogs and later pigs to chemically isolate insulin for the treatment of diabetes in the 1920s. In the 1940s and 1950s, Drs. Jonas Salk and Albert Sabin used nonhuman primates to develop vaccines against the polio virus. Although an estimated 100,000 Rhesus monkeys were killed during the creation of the vaccines, they virtually eradicated polio in the United States and in other developed nations. Additionally, animal studies have enabled physicians to safeguard the population with vaccines against rabies, pertussis, and anthrax. Numerous medications including lithium and penicillin have also been derived from animal studies. Dr. Alfred Blalock used dogs to model physiological shock and developed protocols for cardiovascular resuscitation. These protocols were applied during World War II, saving countless lives. Dr. Blalock would build upon his work and ultimately, perform the first cardiac surgery. In the 1960s, Drs. Albert Starr and Alain Carpenter experimented on dog and pig models to pioneer the field of heart valve replacement and valve prosthetics.

Presently, animal experimentation is conducted to offer new solutions to medical problems ranging from HIV/AIDS to Alzheimer's disease to spinal cord injury.

As illustrated above, many treatments and advances in medicine, including the development and refinement of vaccines, medications, and technological innovations such as the CT scan, were first tested on animals. The reason for such testing historically includes the following: (i) animal models can be environmentally and genetically manipulated to display particular traits under study; (ii) animal models can provide insight into the pathophysiology of disease and help in identifying targets for research; (iii) the safety or toxicity of new treatments can be investigated prior to human trials; and (iv) the efficacy of treatments can be investigated, enabling researchers to abandon or redirect efforts that do not appear to be clinically useful.

Despite the historical and anecdotal evidence proclaiming the benefit of animal research, some recent papers have questioned why the results of animal studies are not replicated consistently in human trials and whether animal experimentation truly translates into clinical benefit for human patients. Possible explanations for this failure to consistently replicate animal study results in human clinical trials include poor methodological standards in animal studies and the lack of uniform reporting of animal data.[1] However, a full discussion on the benefits vs costs of animal research and its translational value in the field of emergency medicine and related fields is beyond the scope of this policy statement.

Moreover, awareness in both the scientific community and general public continues to evolve with respect to animal experimentation and bioethics. Opposition to the practice of vivisection and the use of animals in medical research historically waxes and wanes, but has recently been on the upswing, resulting in the greatly diminished use of animal models in medical schools. The debate involves a continuum of those who support animal testing without limitation to those opposed to animal testing in any form.

Most modern physician-scientists support the responsible use of animal experimentation to further medical and biological knowledge (and not mere curiosity) while minimizing the infliction of pain and suffering of the animal subjects. The first law to protect the use of animals in research was the Cruelty to Animals Act, passed in 1876 by the British Parliament, and aimed at the practice of vivisection. The state of law is constantly evolving to address new technologies and physician-scientists should be vigilant in comporting their research to the latest regulations, guidelines, and ethical frameworks.

The editors of the Journal of Emergencies, Trauma, and Shock (JETS) recognize the potential ethical and legal quagmire of animal experimentation and hereby formulate the following policy for articles that report data or conclusions obtained through the use of animals in research to guide our readers and authors in the development and publication of their research.

## POLICY GUIDELINES ON THE HUMANE AND RESPONSIBLE USE AND CARE OF ANIMALS IN RESEARCH FOR THE JOURNAL OF EMERGENCIES, TRAUMA, AND SHOCK (JETS)

1. From the date of publication of this policy, JETS will not solicit, review, accept, or publish any papers utilizing the data obtained through animal research that are not in accordance with JETS clinical trial registration policy.
2. All authors using data obtained from animal research must submit a statement indicating that their project was reviewed and approved by their institution's formally constituted Institutional Review Board or Ethics Committee.
3. All authors using data obtained from animal research must submit a statement confirming that all animal experiments and/or trials were conducted in full compliance with any and all local, national, international, ethical, and regulatory principles.
4. JETS defines a animal research as any study that prospectively utilizes animal subjects or organisms or the products of those animal subjects or organisms including but not limited to non-human primates, vertebrates, invertebrates, and other organisms in developing disease or physiological models or which otherwise assigns the animal subject to either an intervention or comparison group in order to evaluate the causal relationship between a medical intervention and a health or biomedical outcome/end-point.
5. JETS defines interventions as any medical, surgical, psychological, or sociological procedure, therapy, or treatment, including but not limited to medications, herbal, natural, or home remedies, use of therapies including acupuncture, coining, cupping, and other traditional Eastern remedies, orthopedic or chiropractic manipulation, as well as the use of imaging studies such as X-rays, ultrasound, CT, MRI, or PET scans, and the development of genetic cell lines or any other modality designed or utilized by medical personnel for the advancement of basic science or for use in clinical assessment, decision making, and treatment/disposition.[2]

The editors of JETS unanimously believe that in the realm of animal experimentation, we have the responsibility to promote the humane use and care of animals. We require that all authors register their studies with a clinical trial registry if they have conducted a clinical trial involving animals (e.g., testing a new vaccine on an animal for safety or efficacy as part of a clinical trial), comport their clinical research activities in accordance with JETS policy on clinical trials[3] and, at all times, maintain the highest degree of integrity while conducting research.
